# Novel Two-Dimensional Layered MoSi_2_Z_4_ (Z = P, As): New Promising Optoelectronic Materials

**DOI:** 10.3390/nano11030559

**Published:** 2021-02-24

**Authors:** Hui Yao, Chao Zhang, Qiang Wang, Jianwei Li, Yunjin Yu, Fuming Xu, Bin Wang, Yadong Wei

**Affiliations:** 1 College of Physics and Optoelectronic Engineering, Shenzhen University, Shenzhen 518060, China; yaohui@szu.edu.cn (H.Y.); qiangwang@szu.edu.cn (Q.W.); jianweili@szu.edu.cn (J.L.); xufuming@szu.edu.cn (F.X.); ywei@szu.edu.cn (Y.W.); 2 Laboratory of Optoelectronic Devices and Systems of Ministry of Education and Guangdong Province, College of Physics and Optoelectronic Engineering, Shenzhen University, Shenzhen 518060, China; 3 Beijing Computational Science Research Center, Beijing 100193, China; chaozhang@csrc.ac.cn

**Keywords:** DFT, remarkable optical absorption, superior external quantum efficiency, optoelectronic devices

## Abstract

Very recently, two new two-dimensional (2D) layered semi-conducting materials MoSi${}_{2}$N${}_{4}$ and WSi${}_{2}$N${}_{4}$ were successfully synthesized in experiments, and a large family of these two 2D materials, namely MA${}_{2}$Z${}_{4}$, was also predicted theoretically (Science, 369, 670 (2020)). Motivated by this exciting family, in this work, we systematically investigate the mechanical, electronic and optical properties of monolayer and bilayer MoSi${}_{2}$P${}_{4}$ and MoSi${}_{2}$As${}_{4}$ by using the first-principles calculation method. Numerical results indicate that both monolayer and bilayer MoSi${}_{2}$Z${}_{4}$ (Z = P, As) present good structural stability, isotropic mechanical parameters, moderate bandgap, favorable carrier mobilities, remarkable optical absorption, superior photon responsivity and external quantum efficiency. Especially, due to the wave-functions of band edges dominated by *d* orbital of the middle-layer Mo atoms are screened effectively, the bandgap and optical absorption hardly depend on the number of layers, providing an added convenience in the experimental fabrication of few-layer MoSi${}_{2}$Z${}_{4}$-based electronic and optoelectronic devices. We also build a monolayer MoSi${}_{2}$Z${}_{4}$-based 2D optoelectronic device, and quantitatively evaluate the photocurrent as a function of energy and polarization angle of the incident light. Our investigation verifies the excellent performance of a few-layer MoSi${}_{2}$Z${}_{4}$ and expands their potential application in nanoscale electronic and optoelectronic devices.

## 1. Introduction

Two-dimensional (2D) materials have attracted extensive attention due to their distinctive physical and material properties and the potential application on account of monolayer limit [1,2,3,4,5,6,7,8,9]. As a typical representative, graphene has been widely expected to be a proper material for the preparation of a new generation of nanoelectronic devices due to remarkable high carrier mobility, but its zero bandgap reminds us that it may not be an effective solution [1,10,11]. Transition metal dichalcogenides (TMDCs) possess tunable bandgap, but its comparatively low carrier mobilities is a not neglectable obstacle for practical applications [3,12,13,14]. For example, the carrier mobility of MoS${}_{2}$ is roughly 72 cm${}^{2}$V${}^{-1}$s${}^{-1}$ for electron and 200 cm${}^{2}$V${}^{-1}$s${}^{-1}$ for hole, which are roughly four to six orders of magnitude smaller than graphene and even much lower than those of low-doped Si (1350 cm${}^{2}$V${}^{-1}$s${}^{-1}$ for electron and 480 cm${}^{2}$V${}^{-1}$s${}^{-1}$ for hole). Beyond TMDCs, a large 2D family of transition metal carbides and carbonitrides, called MXenes, has been discovered in recent years [15,16,17]. MXenes are produced by the etching out of the A layers from MAX phases of M${}_{n+1}$AX${}_{n}$, where M is a transition metal, A is mainly a group IIIA or IVA element, and X is C or N with n=1,2,3. So far, more than 60 different pure MXenes have been explored. Their electronic properties, such as band-gap and magnetism, can be tuned by changing the MXene elemental composition and the surface terminations.

Very recently, a new kind of hexagonal 2D MXene, MoSi${}_{2}$N${}_{4}$ and WSi${}_{2}$N${}_{4}$, was successfully synthesized by chemical vapor deposition method with large size up to 15 mm × 15 mm [18]. They show good environmental stability, and even have no structural deformation within six months. Monolayer MoSi${}_{2}$N${}_{4}$ is septuple-atomic-layer structure of N-Si-N-Mo-N-Si-N, which can be view as a MoN${}_{2}$ layer sandwiched by two SiN layers. It exhibits indirect bandgap semiconducting behavior with band-gap roughly equal to 1.94 eV. The elastic modulus is four times that of monolayer MoS${}_{2}$, and electron/hole mobility is also roughly four-to-six times larger than that of monolayer MoS${}_{2}$. In addition, a large family of MA${}_{2}$Z${}_{4}$ is predicted by first-principles calculation, where M represents the elements of IVB, VB, or VIB groups, A represents Si or Ge, and Z represents the elements of VA group. The nanosheets in this family are expected to have wide tunable bandgap and magnetic properties, meaning potential application in electronics, optoelectronics and spintronics.

Motivated by the exciting properties of MoSi${}_{2}$N${}_{4}$ and WSi${}_{2}$N${}_{4}$, some theoretical works have been carried out to further explore the mechanical and physical properties of their family by using the first-principles calculation method [19,20]. The lattice thermal conductivity, piezoelectric and flexoelectric response, and photocatalytic and electronic feature of monolayer MA${}_{2}$Z${}_{4}$ (M = Cr, Mo, W; A = Si, Ge; Z = N, P) were systematically calculated. They show diverse electronic properties from antiferromagnetic metal to half metal and semiconductor with band gaps ranging from 0.31 to 2.57 eV. Monolayer MoSi${}_{2}$N${}_{4}$ and WSi${}_{2}$N${}_{4}$ were predicted to show outstandingly high lattice thermal conductivity of 440 and 500 W/mK, respectively [19]. The piezoelectricity property was calculated for six different configurations of MSi${}_{2}$N${}_{4}$ (M = Mo, W) which are built through translation, mirror and rotation operations. The maximum piezoelectric strain and stress coefficients is 3.53 pm/V and $13.95\times 10^{-10}$ C/m for MoSi${}_{2}$N${}_{4}$, and 2.91 pm/V and $12.17\times 10^{-10}$ C/m for WSi${}_{2}$N${}_{4}$, respectively, which are much larger than those of 2D TMD, metal oxides, III-V semiconductor and Janus TMD [20]. By tuning biaxial in-plane strain to monolayer VSi${}_{2}$P${}_{4}$, a continuous phase transition can be occurred from a ferromagnetic metal to a spin-gapless semiconductor to a ferromagnetic semiconductor to spin-gapless semiconductor to a ferromagnetic half-metal. At the ferromagnetic semiconductor phase, ferromagnetism and piezoelectricity can exist together due to broken inversion symmetry [21]. The van der Waals hetero-structures composed of MoSi${}_{2}$N${}_{4}$ contacted by graphene and NbS${}_{2}$ monolayers were predicted to exhibit ultra-low Schottky barrier height, which can be modulated via the interlayer distance or external electric field [22]. Due to the intrinsic inversion symmetry breaking and strong spin–orbital coupling, remarkable spin-valley coupling in the inequivalent valleys at *K* and $K^{{}^{\prime }}$ points can be found for MoSi${}_{2}$X${}_{4}$ (X = N, P, As). It induces spin-valley coupled optical selection properties, which can be tuned by in-plane strain [23]. Beyond traditional two-level valleys, monolayer MoSi${}_{2}$N${}_{4}$ shows multiple folded valleys, implying an additional intrinsic degree of freedom. The valley-contrasting properties in monolayer MoSi${}_{2}$N${}_{4}$ were discussed by using a three-band low-power Hamiltonian, where each valley and energy band can be selectively controlled [24].

In this paper, we systematically investigate the structural, electronic, optoelectronic and quantum transport properties of monolayer and bilayer MoSi${}_{2}$Z${}_{4}$ (Z = P, As). All these 2D materials possess stable configuration, moderate direct band-gap, high and anisotropic carrier mobilities, large optical absorption coefficient, superior photon responsivity and external quantum efficiency in the visible light region. An optoelectronic device based on monolayer MoSi${}_{2}$Z${}_{4}$ is also built to model the adjustable photocurrent. Our investigation further expands the application prospect of few-layer MoSi${}_{2}$Z${}_{4}$ in nanoelectronics and optoelectronics.

The rest of this paper is organized as follows. In Section 2, the computational methods are briefly introduced. In Section 3, the numerical results of the structural, electronic and optoelectronic properties are presented. In addition, the photocurrent of monolayer MoSi${}_{2}$Z${}_{4}$-based nanodevice is also calculated. In Section 4, a brief summary is presented.

## 2. Numerical Methods

A first-principles calculation is performed by using the Vienna *ab initio* simulation package [25,26] based on the density functional theory (DFT). Both the generalized gradient approximation with a PBE form [27] and the Heyd-Scuseria-Ernzerhof (HSE06) [28] hybrid functional is adopted to calculate the band structures and optical-electronic properties. The energy cutoff and reciprocal k-points are chosen as 500 eV and 16×16×1 in structure relaxation and electronic calculation. A vacuum space of 20 Å perpendicular to the 2D plane is applied to separate the periodic images. The weak vdW interaction between adjacent layers is described by the DFT-D2 functional with Grimme correction [29]. The convergence criteria of force and energy are set to 0.01 eV/Å and $10^{-5}$ eV. To examine the stability of all the structures, PHONOPY code is used to calculate the phonon dispersion curves [30], and *ab initio* molecular dynamics (AIMD) simulation [31] is carried out to examine the total energy evolution at high temperature. To calculate the photocurrent of 2D layered MoSi${}_{2}$Z${}_{4}$ based nanodevice, Nanodcal software is evaluated which is developed based on the combination of DFT and non-equilibrium Green’s function (NEGF-DFT) [32]. In the calculation, norm-conserving pseudopotential, double-zeta polarization basis set and exchange-correlation functional at PBE level are employed.

## 3. Results and Discussion

### 3.1. Structural and Mechanical Properties of Few-Layer MoSi${}_{2}$Z${}_{4}$ (Z = P, As)

**Figure 1:**
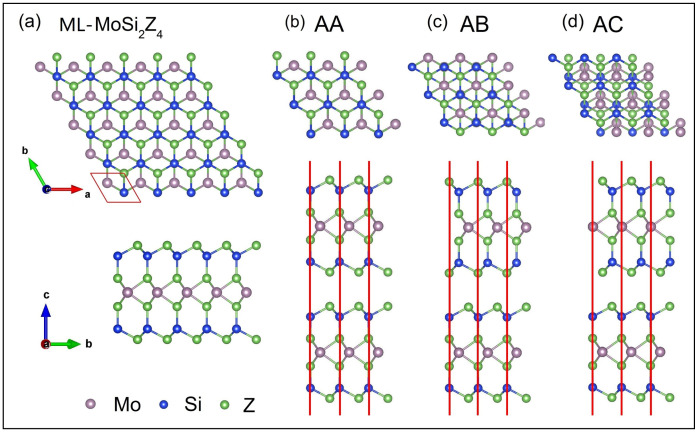
(**a**) Schematic structure of monolayer MoSi${}_{2}$Z${}_{4}$ (Z = P, As) from top view and side view. The parallelogram indicates its primitive cell. (**b**) AA, (**c**) AB, (**d**) AC stacking patterns of bilayer MoSi${}_{2}$Z${}_{4}$.

Figure 1 shows the optimized schematic structures of monolayer (a) and bilayer (b–d) MoSi${}_{2}$Z${}_{4}$ from top view and side view, where Z = P, As. Monolayer MoSi${}_{2}$Z${}_{4}$ is constructed from septuple atomic layers of Z–Si–Z–Mo–Z–Si–Z, which can be viewed as a MoZ${}_{2}$ layer sandwiched by two SiZ layers. It presents A–B stacked hexagonal lattice from the top view, and its primitive cell includes one Mo atom, two Si atoms and four Z atoms as labeled by the parallelogram in Figure 1a. The lattice parameters a=b= 3.470 Å and 3.620 Å for Z = P and As, respectively, which are well coincident with those predicted in previous work [18]. Figure 1b–d present three most likely stacking patterns of bilayer MoSi${}_{2}$Z${}_{4}$, namely AA, AB and AC, where the Si atoms in the lower layer are aligned with the Si, Z, and Mo atoms in the upper layer, respectively. The relaxed lattice parameters *a* and interlayer distances *d* are listed in Table 1 for each stacking pattern and two kinds of Z atoms. We find that the interlayer distance of AB stacking is the smallest compared to the other two stacking patterns for both MoSi${}_{2}$P${}_{4}$ and MoSi${}_{2}$As${}_{4}$.

Firstly, we check the stability of monolayer and bilayer MoSi${}_{2}$Z${}_{4}$ before further studying their physical properties. For monolayer MoSi${}_{2}$Z${}_{4}$, the cohesive energy is calculated by

(1) $$E_{c}=\left(E_{Mo}+2E_{Si}+4E_{Z}-E_{MoSi_{2}Z_{4}}\right)/7,$$

where $E_{Mo}$, $E_{Si}$, $E_{Z}$ and $E_{MoSi_{2}Z_{4}}$ are total energies of isolated Mo atom, Si atom, Z atom and a primitive cell of MoSi${}_{2}$Z${}_{4}$. The calculated cohesive energies are 6.089 eV/atom for MoSi${}_{2}$P${}_{4}$ and 5.475 eV/atom for MoSi${}_{2}$As${}_{4}$. They are smaller than that of graphene (7.46 eV/atom), while larger than those of MoS${}_{2}$ (4.98 eV/atom) and phosphorene (3.30 eV/atom) [33,34,35] indicting proper stability. For bilayer MoSi${}_{2}$Z${}_{4}$, the stability is generally measured by the binding energy defined as

(2) $$E_{b}=E_{BL}-2E_{ML},$$

where $E_{BL}$ and $E_{ML}$ stand for total energies of bilayer and monolayer MoSi${}_{2}$Z${}_{4}$, respectively. As listed in Table 1, the binding energies are negative for all the bilayer MoSi${}_{2}$Z${}_{4}$, and the AB stacking has the smallest value indicting the most stable stacking patten. Thus, we only focus on the AB stacking pattern for the bilayer MoSi${}_{2}$Z${}_{4}$ in the rest of this paper.

**Figure 2:**
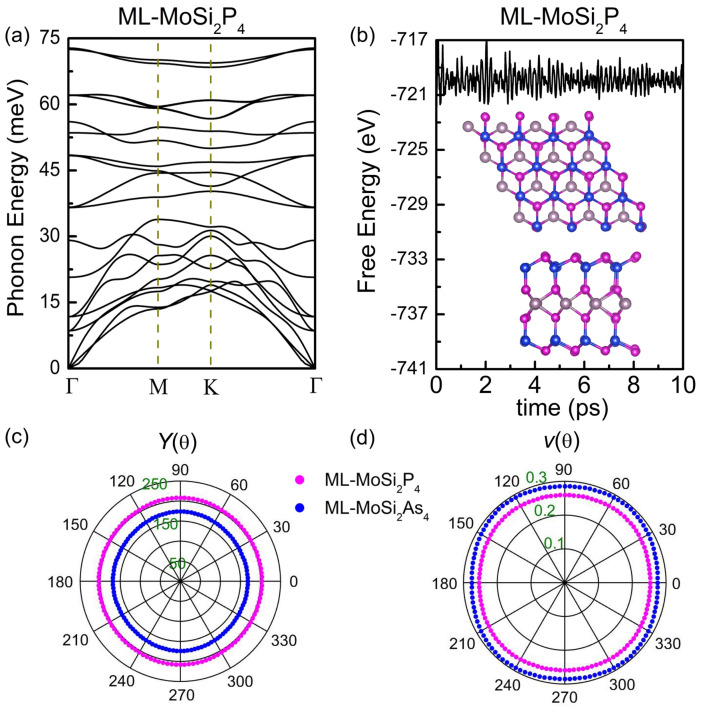
(**a**) Phonon dispersion curves and (**b**) total energy variation at 300 K of monolayer MoSi${}_{2}$P${}_{4}$. Inset in (**b**) shows the top view and side view of a snapshot at 10 ps. (**c**) Young’s modulus Y(θ) and (**d**) Poisson’s ratio ν(θ) of monolayer MoSi${}_{2}$P${}_{4}$ (purple curve) and MoSi${}_{2}$As${}_{4}$ (blue curve) along arbitrary in-plane directions.

**Figure 3:**
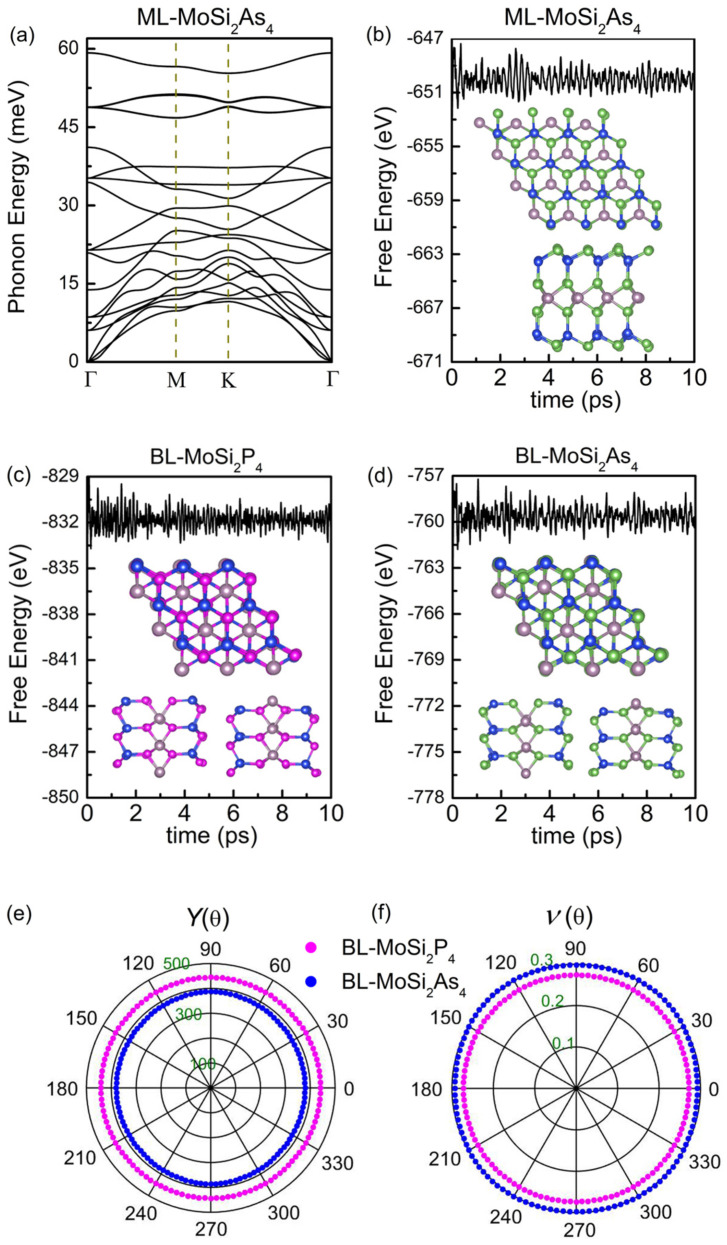
(**a**) Phonon band dispersion curves of the monolayer MoSi${}_{2}$As${}_{4}$. Variations of total energy at 300 K as functions of time for (**b**) monolayer MoSi${}_{2}$As${}_{4}$, (**c**) bilayer MoSi${}_{2}$P${}_{4}$, (**d**) bilayer MoSi${}_{2}$As${}_{4}$. (**e**) Young’s modulus Y(θ) and (**f**) Poisson’s ratio ν(θ) of bilayer MoSi${}_{2}$P${}_{4}$ (purple curve) and MoSi${}_{2}$As${}_{4}$ (blue curve) along arbitrary in-plane directions.

Next, the phonon dispersion spectrums of monolayer MoSi${}_{2}$Z${}_{4}$ are calculated to examine their dynamic stability. Figure 2a presents the phonon dispersion spectrum of monolayer MoSi${}_{2}$P${}_{4}$. The low-frequency band near Γ point is roughly linear and there is no imaginary modes in the Brillouin zone, which indicates monolayer MoSi${}_{2}$P${}_{4}$ is dynamically stable. An AIMD simulation is performed at 300 K to further examine the thermal stability of the structure by employing a 4×4 supercell. As shown in Figure 2b, the total energy of monolayer MoSi${}_{2}$P${}_{4}$ oscillates slightly in the vicinity of −720 eV for a long time without decay. Neither bond-breaking nor geometry reconstruction appears in the structure at 10 fs indicating thermal stability of monolayer MoSi${}_{2}$P${}_{4}$ at room temperature. Similar phonon dispersion spectrums and total energy evaluations are also obtained for all the other monolayer and bilayer structures, as shown in Figure 3.

Finally, we examine the mechanical properties of all the structures under external force by calculating elastic constants $C_{ij}$. As listed in Table 2, the Born criteria $C_{11}C_{22}-C_{12}^{2}>0$ and $C_{66}>0$ are both satisfied for the monolayer and bilayer MoSi${}_{2}$Z${}_{4}$ meaning their mechanical stability [36]. Based on $C_{i,j}$, Young’s modulus Y(θ) and the Poisson’s ratio ν(θ) along the in-plane angle θ and the layer modulus γ are also calculated. Y(θ) indicates the reciprocal of the response of strain to stress along a specific direction along θ in the 2D plane. ν(θ) is the ratio of the absolute value of transverse normal strain to axial normal strain. γ represents the resistance of the 2D surface to stretching, and thus is independent of θ. These physical quantities can be calculated by the following formulas [37]

$$Y\left(\theta \right)=\frac{C_{11}C_{22}-C_{12}^{2}}{C_{11}\sin^{4}\theta +A\sin^{2}\theta \cos^{2}\theta +C_{22}\cos^{4}\theta },$$

$$\nu \left(\theta \right)=\frac{C_{12}\sin^{4}\theta -B\sin^{2}\theta \cos^{2}\theta +C_{12}\cos^{4}\theta }{C_{11}\sin^{4}\theta +A\sin^{2}\theta \cos^{2}\theta +C_{22}\cos^{4}\theta },$$

$$\gamma =\frac{1}{4}\left(C_{11}+C_{22}+2C_{12}\right),$$

in which $A=\left(C_{11}C_{22}-C_{12}^{2}\right)/C_{66}-2C_{12}$ and $B=C_{11}+C_{22}-\left(C_{11}C_{22}-C_{12}^{2}\right)/C_{66}$. Figure 2c,d show the Y(θ) and ν(θ) of monolayer MoSi${}_{2}$Z${}_{4}$. Y(θ) is isotropic and ν(θ) is roughly isotropic for both monolayers. Y(θ) of MoSi${}_{2}$P${}_{4}$ is larger than that of MoSi${}_{2}$As${}_{4}$, while ν(θ) of the former is smaller to that of the latter. This means monolayer MoSi${}_{2}$As${}_{4}$ is easier to deform under in plane external force than monolayer MoSi${}_{2}$P${}_{4}$. It is reasonable because the As–Mo and As–Si bonds are longer and deformable than the P–Mo and P–Si bonds. Similarly, γ of MoSi${}_{2}$P${}_{4}$ is larger than that of MoSi${}_{2}$As${}_{4}$. *Y* and γ of monolayer MoSi${}_{2}$Z${}_{4}$ are slightly smaller than that of monolayer graphene (340 N/m and 215.9 N/m) and BN (318 N/m and 177.0 N/m) [38], while comparable to those of SiC (179.7 N/m and 116.5 N/m) [38] and monolayer PC${}_{3}$ (180.4 N/m and 102.1 N/m) [39] implying their similar mechanical response. In terms of bilayer MoSi${}_{2}$Z${}_{4}$, both Y(θ) and γ are nearly two times as those of monolayer MoSi${}_{2}$Z${}_{4}$ (see Table 2 and Figure 3). Such behavior is physically reasonable and in good accordance with that of multilayer graphene [38] and PC${}_{3}$ [39]. The calculated moduli indicate that few-layered MoSi${}_{2}$Z${}_{4}$ are stretchable and flexible as most of the other common 2D materials, indicting potential application in flexible electronic devices.

### 3.2. Electronic Properties of Few-Layer MoSi${}_{2}$Z${}_{4}$

**Figure 4:**
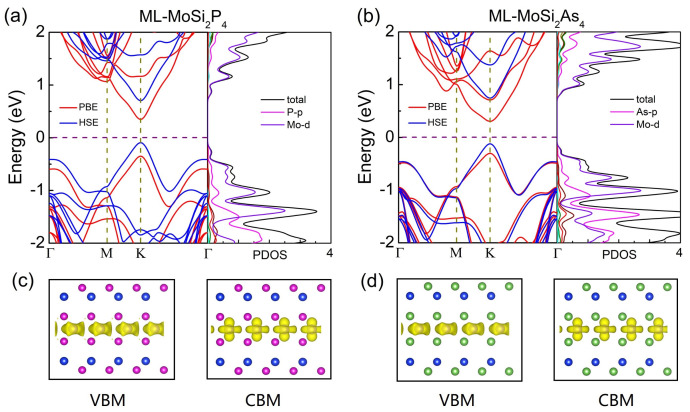
The electronic band structure and projected density of states (PDOS) of monolayer (**a**) MoSi${}_{2}$P${}_{4}$ and (**b**) MoSi${}_{2}$As${}_{4}$. (**c**,**d**): Corresponding charge distribution at valence band maximum (VBM) and conduction band minimum (CBM) dominated by the *d* orbital of Mo atoms in the middle layer.

**Figure 5:**
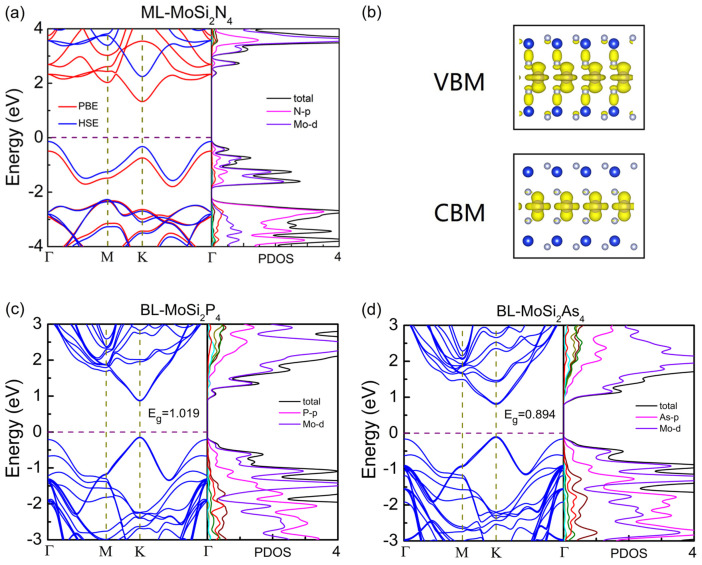
(**a**) The electronic band structure and PDOS of monolayer MoSi${}_{2}$N${}_{4}$, (**b**) Corresponding charge distribution at VBM and CBM of monolayer MoSi${}_{2}$N${}_{4}$. The electronic band structure and PDOS of bilayer (**c**) MoSi${}_{2}$P${}_{4}$ and (**d**) MoSi${}_{2}$As${}_{4}$.

Figure 4a,b show the band structure and projected density of states (PDOS) of monolayer MoSi${}_{2}$P${}_{4}$ and MoSi${}_{2}$As${}_{4}$ based on PBE and HSE06 exchange-correlation functionals. For each configuration, the band structure based on the PBE exchange-correlation functional is similar to that based on the HSE06 functional except the smaller bandgap. Both structures show a direct bandgap, and both conduction band minimum (CBM) and valence band maximum (VBM) locate at K point. This is different from monolayer MoSi${}_{2}$N${}_{4}$, whose CBM sits K point while VBM locates at Γ point, presenting indirect band-gap semiconducting behavior (Ref. [18], also see Figure 5a). To get more insight into this difference, PDOS and charge distribution at VBM and CBM are plotted in Figure 5c,d. For monolayer MoSi${}_{2}$P${}_{4}$ and MoSi${}_{2}$As, both CBMs and VBMs are mainly originated from the *d* orbitals of Mo atoms which locate in the middle layer of the structures. While, for monolayer MoSi${}_{2}$N${}_{4}$, VBM is dominated by both *d* orbital of Mo atoms and *p* orbital of Z atom (see Figure 5b). Due to the orbital hybridization, an obvious extension of VBM from the middle Mo atoms to beside the Z atoms occurs, which is much different from the charge distribution of VBMs in MoSi${}_{2}$P${}_{4}$ and MoSi${}_{2}$As${}_{4}$. This is reasonable because the N–Mo bonds are shorter than the P–Mo and As–Mo bonds, and thus the orbital hybridization is more likely to happen in MoSi${}_{2}$N${}_{4}$.

In terms of bilayer MoSi${}_{2}$Z${}_{4}$, similar direct bandgap semiconducting behavior to monolayer MoSi${}_{2}$Z${}_{4}$ are obtained, where both CBM and VBM locate at K points (see Figure 5c,d). The bandgap of bilayer MoSi${}_{2}$Z${}_{4}$ changes very little in comparison to that of monolayer MoSi${}_{2}$Z${}_{4}$ (see Table 1). The independence of bandgap with the number of layers can be attributed to the orbital shield. Because the states at CBM and VBM are dominated by the *d* orbital of Mo atoms, they are effectively screened inside the monolayer MoSi${}_{2}$Z${}_{4}$ because the Mo atoms located in the middle layer of seven atomic layers. For bilayer MoSi${}_{2}$Z${}_{4}$, the rather weak interlayer vdW interaction makes the Mo atoms at the up layer and those at the down layer have nothing to do with each other, and thus the band gap is very close to that of the monolayer. Similar layer number independent bandgap behavior has also been found in layered 2D KAgSe [40]. The layer number independent electronic properties provide enormous convenience and less difficulty in experimental fabrication of finite layer MoSi${}_{2}$Z${}_{4}$-based electronic devices.

### 3.3. Carrier Mobilities of Few-Layer MoSi${}_{2}$Z${}_{4}$

Carrier mobility is an important factor to describe the transport ability of electronic and optoelectronic materials, which can be evaluated by using the deformation potential method as follows [41,42],

(3) $$\mu =\frac{eћC}{k_{B}Tm^{*}m_{d}E_{DP}^{2}},$$

where *T* is the temperature and equal to 300 K in this calculation; $m^{*}=\pm {ћ}^{2}\left(d^{2}E_{k}/dk^{2}\right)$ is the effective mass of electrons and holes depending on the change of energy with wave vector *k* along different transport directions; $m_{d}$ is the averaged effective mass defined as $m_{d}=\sqrt{m_{x}^{*}m_{y}^{*}}$; $C=\left(\partial^{2}E/\partial^{2}\varepsilon \right)/S_{0}$ is the elastic modulus related to the change of total energy with strain along different directions; $E_{DP}=dE_{edge}/d\varepsilon$ is the deformation potential constant given by the change rate of band edges with strain. The calculated carrier mobilities and corresponding parameters of layered MoSi${}_{2}$Z${}_{4}$ are summarized in Table 3.

Three pieces of information can be obtained from Table 3. Firstly, the carrier mobility of holes is roughly three to four times larger than that of electrons for both monolayer and bilayer MoSi${}_{2}$Z${}_{4}$ along with both x and y directions, which mainly attributes to the smaller deformation potential constant $E_{DP}$ of holes. This difference of carrier mobilities can effectively facilitate the spatial separation of electrons and holes, which reduces the recombination probability of photo-excited carriers and suggests satisfactory performances for nanoscale electronic and optoelectronic devices. Secondly, the carrier mobilities of bilayer MoSi${}_{2}$Z${}_{4}$ are largely improved in contrast to those of monolayer MoSi${}_{2}$Z${}_{4}$ due to the roughly doubled elastic modulus *C*. Similar properties were also found for MXs [43]. Thirdly, the carrier mobilities of MoSi${}_{2}$P${}_{4}$ are slightly higher than that of MoSi${}_{2}$As${}_{4}$ for both monolayer and bilayer structures, which are also independent of carrier types and directions. Especially, these carrier mobilities are relatively high, which are much larger than those of MoS${}_{2}$ (200–500 cm${}^{2}$V${}^{-1}$s${}^{-1}$) [44] and even comparable to those of black phosphorene [42] indicating potential application in 2D electronic devices.

### 3.4. Optical Absorption Spectrums of Layered MoSi${}_{2}$Z${}_{4}$

Monolayer and bilayer MoSi${}_{2}$Z${}_{4}$ with direct band gaps about 0.85–1.0 eV exhibit potential application for visible–light solar harvesting/utilizing techniques or making narrow-gap semiconductor devices. Recent studies revealed that such narrow band gap materials are good candidates of infrared photodetectors, such as phosphorus carbides and black arsenic phosphorus [6,45,46]. Thus, we further investigate the optoelectronic performance of few-layer MoSi${}_{2}$Z${}_{4}$ by calculating the absorption coefficient as follows [47,48]

(4) $$\alpha \left(\omega \right)=\sqrt{2}\omega \sqrt{\sqrt{\varepsilon_{1}^{2}\left(\omega \right)+\varepsilon_{2}^{2}\left(\omega \right)}-\varepsilon_{1}\left(\omega \right)},$$

where *c*, ω, $\varepsilon_{1}\left(\omega \right)$ and $\varepsilon_{2}\left(\omega \right)$ stands for the light velocity, frequency of incident light, real part and imaginary part of the frequency-dependent dielectric function, respectively. $\varepsilon_{1}\left(\omega \right)$ and $\varepsilon_{2}\left(\omega \right)$ can be calculated by using the Kramers–Kronig relation and summing all the empty states in the Brillouin zone.

**Figure 6:**
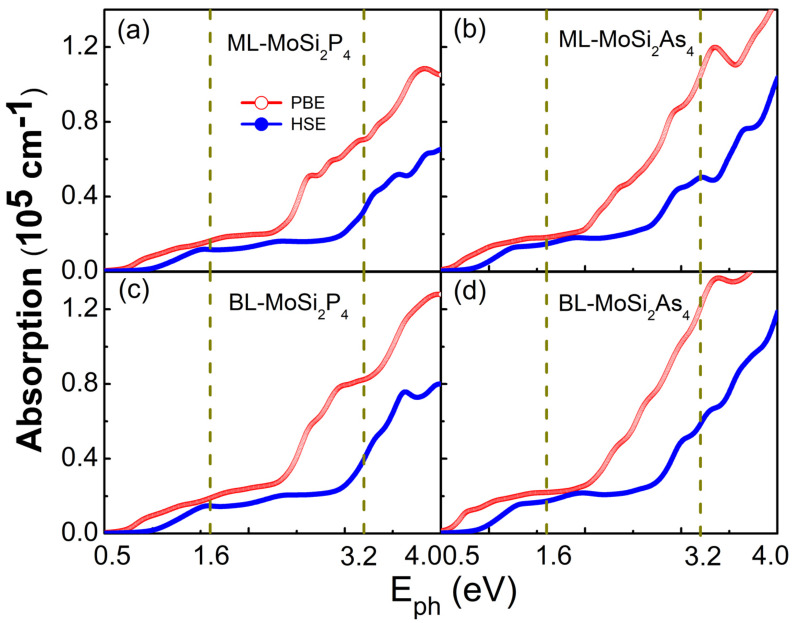
Optical absorption coefficients versus the energy of incident light for 2D (**a**) monolayer MoSi${}_{2}$P${}_{4}$, (**b**) monolayer MoSi${}_{2}$As${}_{4}$, (**c**) bilayer MoSi${}_{2}$P${}_{4}$ and (**d**) bilayer MoSi${}_{2}$As${}_{4}$ based on PBE and HSE06 functionals. For each panel, the polarization vector of incident light is set parallel to the plane, and the two vertical dashed lines indicate the region of visible light.

Figure 6 shows the optical absorption coefficients of monolayer and bilayer MoSi${}_{2}$Z${}_{4}$ based on PBE and HSE06 calculations, where the polarization direction of incident light is parallel to the 2D plane. All the few-layer MoSi${}_{2}$Z${}_{4}$ display very similar and remarkably high absorption coefficients (∼10${}^{5}$ cm${}^{-1}$) in the visible-ultraviolet light region, which agrees well with their similar band gaps as shown in Figure 4. The large absorption is even comparable to that of graphene, phosphorene and MoS${}_{2}$ [6]. The strong optical absorption and broad absorption ranges make layered MoSi${}_{2}$Z${}_{4}$ promising materials for photovoltaic solar cells and optoelectronic devices. Especially, the layer number independence to the bandgap and optical absorption makes the experimental fabrication more convenient of few-layer MoSi${}_{2}$Z${}_{4}$-based 2D optoelectronic devices.

### 3.5. Photocurrent in Monolayer MoSi${}_{2}$Z${}_{4}$ Nanodevice

**Figure 7:**
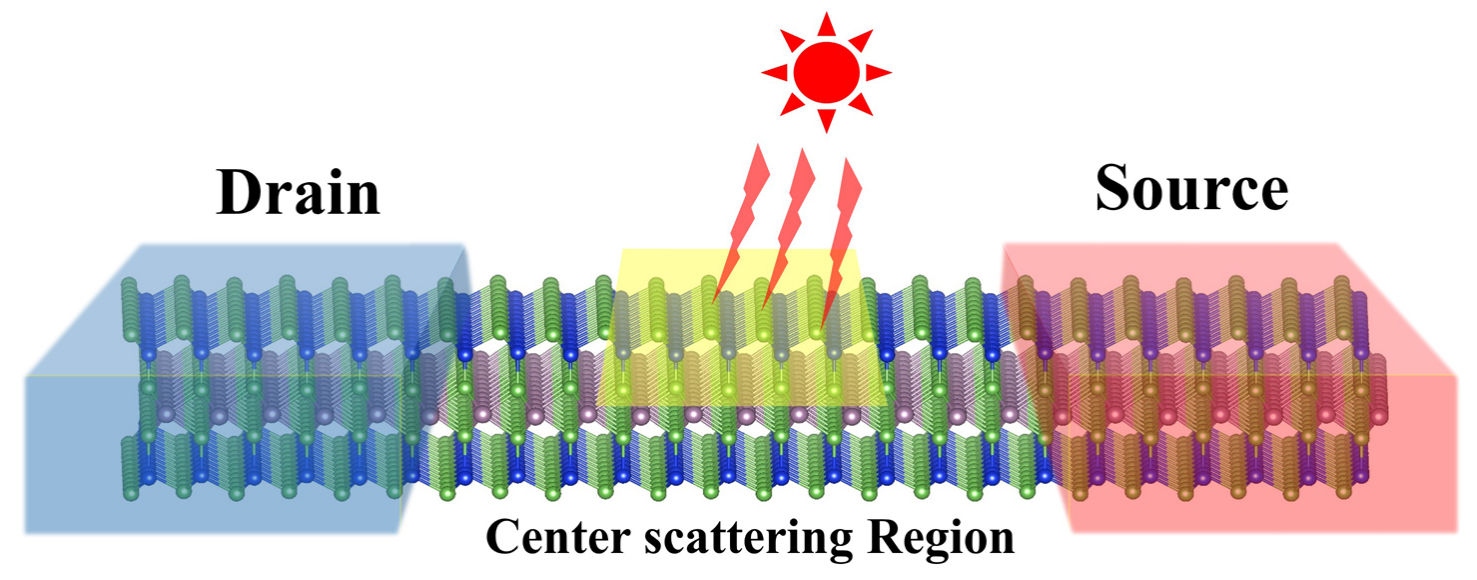
Schematic structure of monolayer MoSi${}_{2}$Z${}_{4}$-based 2D optoelectronic device. The yellow zone in the center scattering region stands for the lighting area. The left blue region and right red region represent the drain and source, respectively.

On account of the similar and excellent optical absorption performance of layered MoSi${}_{2}$Z${}_{4}$, we build a monolayer MoSi${}_{2}$Z${}_{4}$-based two-probe 2D optoelectronic device as shown in Figure 7 and evaluate its photoinduced current. To solve the quantum transport problem in this identical system, the device can be separated into three parts theoretically including a central scattering region and two semi-infinite electrodes. When the incident light energy in the scattering region is larger than the bandgap, the electrons at the valence band can be excited to the conduction band by absorbing photons. When a tiny external bias is applied between the source and the drain, the excited electrons can be driven to produce photocurrent in the system. Note that the potential difference between the left and the right leads should be much smaller than the bandgap of the system to ensure that the detected current in the electrode is completely generated by the light but not bias. The photocurrent flowing into the left probe can be expressed in terms of the NEGF as follows [49,50,51],

(5) $$J_{L}^{ph}=\frac{ie}{h}\int \mathrm{Tr}\left[\Gamma_{L}\left\{G^{<(ph)}+f_{L}\left(E\right)\left(G^{>(ph)}-G^{<(ph)}\right)\right\}\right]dE,$$

where $f_{L}$, $\Gamma_{L}$ and $G^{</>(ph)}$ denotes the Fermi distribution function, line-width function and greater/lesser Green’s function of the two-probe system including electron-photon interaction, respectively.

**Figure 8:**
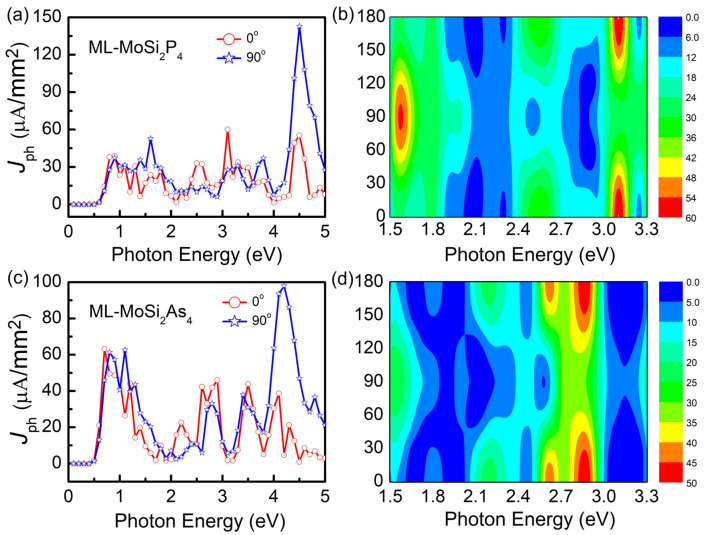
Photocurrent as a function of photon energy with polarization angle θ of the incident light equal to $0^{\circ }$ and $90^{\circ }$ for monolayer (**a**) MoSi${}_{2}$P${}_{4}$ and (**c**) MoSi${}_{2}$As${}_{4}$ nanodevice. (**c**,**d**) show the photocurrent versus photon energy and θ for monolayer (**b**) MoSi${}_{2}$P${}_{4}$ and (**d**) MoSi${}_{2}$As${}_{4}$ nanodevice.

In this calculation, the incident light is perpendicular to the 2D plane, and the angle between polarization direction and transport direction is labeled as θ. Figure 8a,c show the photocurrent versus energy of the linearly polarized light with power density equal to $10^{3}\;\mu W/mm^{2}$ and θ equal to $0^{\circ }$ and $90^{\circ }$. When energy is smaller than 0.5 eV, photocurrent is equal to zero for both MoSi${}_{2}$P${}_{4}$ and MoSi${}_{2}$As${}_{4}$ because the energy is smaller than their band gaps. With further increase of energy, photocurrent appears and oscillates with the energy for both $\theta =0^{\circ }$ and $90^{\circ }$ depending on the detailed behavior of band structures. Photocurrent reaches local maximum in the visible region at $\theta =0^{\circ }$, and in the ultraviolet region at $\theta =90^{\circ }$. To further explore the influence of incident polarization angle θ, photocurrent as a function of θ under different photon energy in the visible light region are evaluated as shown in Figure 6b,d. For both MoSi${}_{2}$P${}_{4}$ and MoSi${}_{2}$As${}_{4}$, the photocurrent is roughly symmetrical with respect to $\theta =90^{\circ }$, and reaches maximums at $\theta =0^{\circ }$ and $\theta =180^{\circ }$. Similar symmetrical distribution of photocurrent with polarization angle was also reported for monolayer KAgSe-based 2D optoelectronic device [40].

The responsivity $R_{ph}$ and external quantum efficiency $\tau_{eqe}$ are generally used to measure the photovoltaic performances, which are defined as

(6) $$R_{ph}=\frac{J_{L}^{ph}}{eF_{ph}}$$

and

(7) $$\tau_{eqe}=R_{ph}\frac{hc}{e\lambda },$$

in which the photon flux $F_{ph}$ stands for the number of incident photons in unit area and unit time. $R_{ph}$ of monolayer MoSi${}_{2}$P${}_{4}$ and MoSi${}_{2}$As${}_{4}$ in the visible light region are 0.060 $AW^{-1}$ and 0.046 $AW^{-1}$, respectively, which are the same order as those of MoS${}_{2}$(0.016 $AW^{-1}$) and monolayer chalcogenides (0.035 $AW^{-1}$ for GeS and 0.075 $AW^{-1}$ for SnS), while two orders higher than that of graphene ($5\times 10^{-4}\;AW^{-1}$) [52]. $\tau_{eqe}$ of monolayer MoSi${}_{2}$P${}_{4}$ and MoSi${}_{2}$As${}_{4}$ in the visible light region can reach 18.60% and 13.33%, respectively, which are comparable to those of KAgSe (17.92%) [40] and monolayer chalcogenides (10.27% for GeS and 22.01% for SnS) [53]. In addition, $R_{ph}$ and $\tau_{eqe}$ of monolayer MoSi${}_{2}$Z${}_{4}$ are greatly increased within the whole light region, ie, 0.143 $AW^{-1}$ and 64.26% for MoSi${}_{2}$P${}_{4}$, 0.098 $AW^{-1}$ and 41.16% for MoSi${}_{2}$As${}_{4}$. Here, it is worth mentioning that the above values of photon responsivity $R_{ph}$ and external quantum efficiency $\tau_{eqe}$ are all calculated theoretically based on the computational models. It is hoping that there will be more experimental results to support in the future. Once again, these ideal performances of MoSi${}_{2}$Z${}_{4}$ suggest their powerful potential application in optoelectronic and photovoltaic devices.

## 4. Conclusions

Recently synthesized 2D semiconductors MoSi${}_{2}$N${}_{4}$ and WSi${}_{2}$N${}_{4}$ exhibit prominent material and physical properties, including remarkable stability, high strength and large carrier mobility, which also inspires increasing theoretical researches to further explore the physical properties of their family MA${}_{2}$Z${}_{4}$. First principle calculations indicate that MA${}_{2}$Z${}_{4}$ materials possess wide tunable band gaps, magnetic properties and valley-contrasting properties, indicating potential applications in electronics, optoelectronics, spintronics and valleytronics. In this case, we investigated the electronic and photoelectrical properties of monolayer and bilayer 2D MoSi${}_{2}$Z${}_{4}$ (Z = P, As) by using the first-principles calculation method. Firstly, the structural, dynamic, thermal and mechanical stabilities of the few-layer MoSi${}_{2}$Z${}_{4}$ were numerically verified. Secondly, both monolayer and bilayer MoSi${}_{2}$Z${}_{4}$ show direct bandgap semiconducting behavior, which is different from MoSi${}_{2}$N${}_{4}$ with indirect bandgap. Moreover, the band gaps of layered MoSi${}_{2}$Z${}_{4}$ are roughly independent of the number of layers due to effective screening to the atomic orbital of Mo atoms. Thirdly, monolayer and bilayer MoSi${}_{2}$Z${}_{4}$ show high carrier mobilities and remarkable optical absorption coefficients. Monolayer MoSi${}_{2}$Z${}_{4}$-based optoelectronic device displays large photon responsivity and external quantum efficiency. All these appealing properties make MoSi${}_{2}$Z${}_{4}$ promising candidates for application in electronic and optoelectronic devices.

## Figures and Tables

**Table 1:** Lattice constants *a*, interlayer distance *d*, cohesive energy $E_{c}$, binding energy $E_{b}$ and band gap of few-layer MoSi${}_{2}$Z${}_{4}$(Z = P, As).

System	Monolayer	Bilayer MoSi${}_{2}$P${}_{4}$			Monolayer	Bilayer MoSi${}_{2}$As${}_{4}$		
Patterns	MoSi${}_{2}$P${}_{4}$	AA	AB	AC	MoSi${}_{2}$As${}_{4}$	AA	AB	AC
$a(\overset{˚}{A})$	3.470	3.449	3.450	3.450	3.620	3.581	3.583	3.583
$d(\overset{˚}{A})$	—	3.850	3.075	3.081	—	3.825	3.108	3.112
$E_{c}\left(eV\right)$	−6.089	—	—	—	−5.475	—	—	—
$E_{b}\left(eV\right)$	—	−3.536	−3.614	−3.613	—	−4.272	−4.385	−4.384
Bandgap(eV)	1.015	0.994	1.019	1.021	0.891	0.888	0.894	0.894

**Table 2:** The calculated elastic constants $C_{ij}$, Young’s modulus *Y* and Poisson’s ratio ν along the *x* (θ=0) and *y* (θ=π/2) directions, layer modulus γ for monolayer (ML-) and bilayer (BL-) MoSi${}_{2}$Z${}_{4}$ (Z = P, As).

Type	$C_{11}\left(N/m\right)$	$C_{22}\left(N/m\right)$	$C_{12}\left(N/m\right)$	$C_{66}\left(N/m\right)$	$Y_{x}\left(N/m\right)$	$Y_{y}\left(N/m\right)$	$\nu_{x}$	$\nu_{y}$	γ(N/m)
ML-MoSi${}_{2}$P${}_{4}$	217.70	222.65	56.35	80.67	203.43	208.06	0.253	0.259	138.26
BL-MoSi${}_{2}$P${}_{4}$	476.59	479.76	130.42	173.09	441.14	444.07	0.272	0.274	304.30
ML-MoSi${}_{2}$As${}_{4}$	182.38	188.67	52.01	65.18	168.04	173.84	0.276	0.285	118.77
BL-MoSi${}_{2}$As${}_{4}$	415.86	423.43	124.00	145.93	379.54	386.45	0.293	0.298	271.82

**Table 3:** The effective mass $m^{*}$, elastic modulus $C_{2D}$, deformation potential constant $E_{DP}$, and carrier mobility μ along x and y directions for monolayer and bilayer MoSi${}_{2}$Z${}_{4}$ at 300 K.

System	CarrierType	$m^{*}/m_{0}$	$C_{2D}$ (Nm${}^{-1})$	$E_{\mathrm{DP}}$ (eV)	μ (cm${}^{2}$V${}^{-1}$s${}^{-1})$
ML-MoSi${}_{2}$P${}_{4}$	e(x)	0.325	214.88	6.82	828.76
	e(y)	0.415	218.74	6.28	778.90
	h(x)	0.339	214.88	3.43	3171.83
	h(y)	0.430	218.74	3.65	2131.78
BL-MoSi${}_{2}$P${}_{4}$	e(x)	0.313	481.13	6.94	1919.84
	e(y)	0.403	484.88	6.40	1759.76
	h(x)	0.344	481.13	2.99	8652.25
	h(y)	0.435	481.88	3.55	4860.34
ML-MoSi${}_{2}$As${}_{4}$	e(x)	0.499	178.40	4.05	823.19
	e(y)	0.640	178.37	3.76	743.11
	h(x)	0.419	178.40	3.04	2093.38
	h(y)	0.524	178.37	3.16	1552.98
BL-MoSi${}_{2}$As${}_{4}$	e(x)	0.496	432.14	4.19	1855.52
	e(y)	0.659	432.12	3.88	1629.69
	h(x)	0.425	432.14	2.79	5905.10
	h(y)	0.528	432.12	2.77	4819.97

## Data Availability

The data presented in this study are available on request from the corresponding author.
